# Real‐Time Targeted Slice Reacquisition for Motion‐Corrupted Fetal Diffusion MRI

**DOI:** 10.1002/mrm.70484

**Published:** 2026-07-10

**Authors:** Jordina Aviles Verdera, Sara Neves Silva, Susanne Schulz‐Heise, Sarah McElroy, Mary Rutherford, Joseph V. Hajnal, Raphael Tomi‐Tricot, Jana Hutter

**Affiliations:** ^1^ Imaging Physics and Engineering Research Department School of Biomedical Engineering and Imaging Sciences, King's College London London UK; ^2^ Institute for Information Processing Leibniz University Hannover Hannover Germany; ^3^ CAIMed, L3S Research Center Hannover Germany; ^4^ Radiology Institute, Uniklinikum Erlangen Erlangen Germany; ^5^ Early Life Imaging Department, School of Biomedical Engineering and Imaging Sciences King's college London UK; ^6^ MR Research Collaborations Siemens Healthcare Limited Camberley UK; ^7^ L3S Research Center Hannover Germany

**Keywords:** artifact detection, brain segmentation, fetal diffusion MRI, real‐time reacquisition

## Abstract

**Purpose:**

To develop and evaluate a real‐time framework for targeted slice‐level reacquisition in fetal diffusion MRI.

**Methods:**

A DW‐SE‐EPI sequence was modified to support independent diffusion preparation per slice. A pathology‐robust nnU‐Net segmentation network, updated from a previously published baseline (V1) using pathological fetal cases and physics‐inspired augmentation (V2), and intensity‐based artifact detection drive automated reacquisition prioritization. Retrospective validation was performed on 60 cases across three cohorts at two field strengths and prospective deployment in 14 cases at two clinical sites.

**Results:**

Segmentation V2 significantly outperformed V1 across all cohorts (p<0.001), with largest gains at b>0 s mm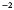 (median DSC gain +0.87 at 1.5 T). Blackout detection achieved F1 = 0.81 ± 0.29 at T = 0.35. Prospective real‐time deployment confirmed clinical feasibility at both field strengths.

**Conclusion:**

Real‐time slice‐level fetal diffusion reacquisition is demonstrated for the first time, opening a pathway toward routine quantitative fetal diffusion MRI in research and clinical settings.

## 1 Introduction

Despite this potential, fetal dMRI remains, however, technically highly challenging. The most significant limitation arises from unpredictable fetal motion, which includes bulk body movements, head rotations, swallowing, and hiccups, compounded by maternal breathing and uterine contractions. These motion sources compromise diffusion‐weighted signal stability and cause a range of artifacts, including slice misalignments and complete signal dropout during diffusion encoding. While conventional single‐shot spin‐echo echo‐planar imaging (EPI) is fast enough to partially mitigate through‐plane motion, it remains highly sensitive to signal loss and geometric distortion. Furthermore, because diffusion modelling and analysis require multiple volumes with different gradient directions or b‐values, even modest motion accumulates across repetitions, progressively degrading data quality.

Fetal Diffusion MRI (dMRI) provides unique insights into the microstructure of the developing brain, supporting both clinical and research applications. By sensitizing the MR signal to the diffusion of water molecules by varying gradients, parametrized by their strength (b‐value) and direction (b‐vector), dMRI yields insights into tissue microstructure and connectivity‐essential for understanding early brain development and detecting deviations from typical trajectories. Clinically, it plays a vital role in cases of suspected pathology such as ventriculomegaly, agenesis of the corpus callosum, neuronal migration disorders, and congenital infections [1]. From a research perspective, it has enabled, for example, the characterization of normative microstructural development [2] and the establishment of in‐vivo atlases of structural connectivity during gestation [3]. The combination of clinical utility and scientific importance underscores the central role of dMRI in advancing prenatal neuroimaging.

Current clinical practice relies on the acquisition of multiple repetitions with identical diffusion preparation, followed by offline quality control and visual inspection/manual selection of acceptable volumes. This approach is, however, highly inefficient and subjective, prolonging scan times and leading to a substantial proportion of discarded data. In difficult cases, particularly in the presence of pathology, an insufficient number of good‐quality volumes may remain, limiting diagnostic value.

A variety of retrospective methods have been developed to overcome these challenges. For structural fetal MRI, slice‐to‐volume reconstruction has been extensively reviewed [4]. For diffusion MRI specifically, analogous approaches including scattered slice reconstruction [5], model‐based reconstruction combining motion correction with higher‐order diffusion signal estimation [6], and more recently comprehensive multi‐shell correction frameworks [7] have demonstrated the feasibility of producing volumetric fetal dMRI data from motion‐corrupted acquisitions. However, these methods require full datasets and offline processing; they cannot prevent information loss when slices are corrupted during acquisition, nor provide feedback during the scan to guide reacquisition.

Another limitation of existing fetal dMRI frameworks is their limited robustness to pathology. Most segmentation networks have been trained predominantly on healthy cohorts, yet fetal MRI is frequently performed in cases of suspected pathology‐including hemorrhage, malformations, and ventriculomegaly‐where such models often fail, reducing the reliability of downstream processing.

In contrast, real‐time detection and correction of motion and artifacts could substantially improve both efficiency and reliability. Real‐time methods are well established in other areas of MRI, such as cardiac imaging [8, 9] or adult brain imaging [10, 11, 12, 13], where motion navigators and prospective correction schemes are routinely used. Analogous solutions for fetal dMRI have not yet been realized, largely due to the complexity of unpredictable fetal motion. A previous method recently published, HERON [14], introduced real‐time brain segmentation and artifact detection for fetal dMRI; however, its robustness to pathologies and severe drop‐outs was limited, and, importantly, it relied on the reacquisition of entire volumes rather than individual corrupted slices.

### 1.1 Contributions

In this work, we address key limitations in fetal dMRI through a unified framework for robust and efficient acquisition and analysis. First, we retrain an nnU‐Net‐based brain segmentation model on a curated dataset of fetal cases with moderate‐to‐severe pathology, augmented with physics‐informed simulations of diffusion artifacts (e.g., slice dropouts), yielding robust generalization to challenging clinical scenarios. Second, we replace volume‐wise diffusion preparation with a slice‐level strategy, enabling selective reacquisition of corrupted slices and improving acquisition efficiency. Third, we integrate the pipeline with a real‐time computing framework using a direct scanner‐GPU optical link and the FIRE framework, enabling immediate segmentation, artifact detection, and on‐the‐fly prioritized slice reacquisition.

Together, these contributions establish a framework for pathology‐robust segmentation with real‐time detection and slice‐level reacquisition in fetal dMRI. We demonstrate improved segmentation accuracy across three challenging retrospective cohorts and show the feasibility of prospective real‐time reacquisition in phantom and in vivo experiments across two sites and two field strengths. Beyond fetal neuroimaging, slice‐level diffusion preparation provides a foundation for motion‐tolerant imaging of other moving organs (e.g., placenta, uterus, liver, bowel). Ultimately, this framework opens a pathway toward routine quantitative fetal dMRI in both research and clinical settings.

## 2 Methods

**FIGURE 1:**
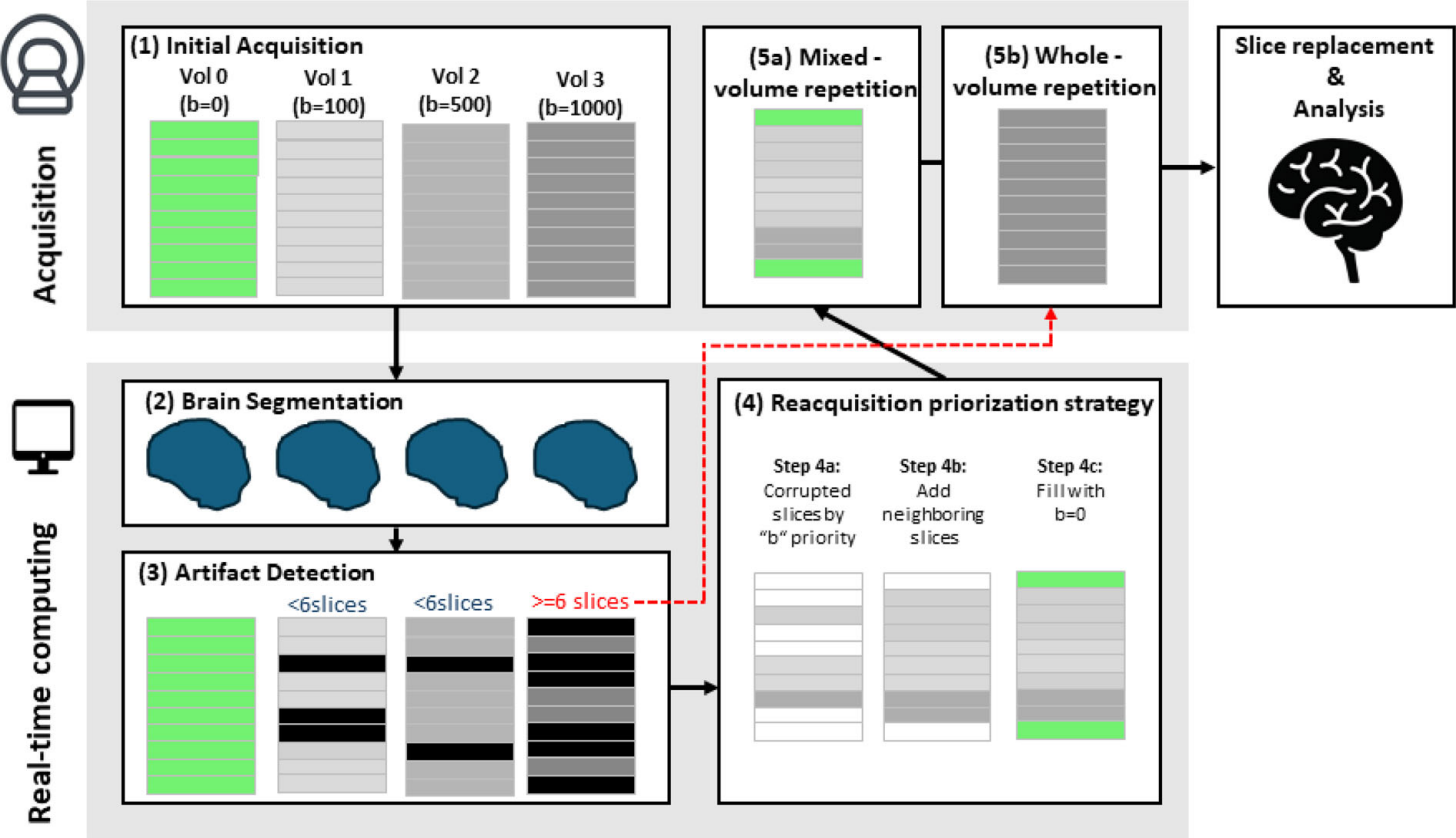
Overview of the proposed strategy consisting of (step 1) initial Diffusion‐Weighted MRI acquisition, (step 2) brain segmentation, (step 3) artifact detection, and (step 4) reacquisition prioritization strategy followed by reacquisition (step 5). Steps 2–4 are performed on a high‐performance computer connected to the MRI host computer. The mixed slice‐level reacquisition and subsequent analysis enable slice‐level correction and diffusion reconstruction.

The entire proposed pipeline is depicted in Figure 1. Following the initial dMRI acquisition (step 1), the fetal brain is segmented in real‐time across all acquired volumes (step 2). Artifact detection is then performed on a slice‐by‐slice basis to identify motion‐corrupted slices within each volume (step 3). After this analysis, a reacquisition prioritization strategy (step 4) constructs a mixed diffusion‐preparation volume containing corrupted slices, which is then reacquired (step 5a). Volumes with extensive motion corruption (≥6 corrupted slices) are instead flagged for conventional whole‐volume repetition (step 5b).

### 2.1 Slice‐Level Diffusion Reacquisition Pipeline

#### 2.1.1 Step 1: dMRI Acquisition

Fetal dMRI data is acquired using a single‐shot DW‐SE‐EPI sequence. In the here‐performed prospective experiments, a typical IVIM acquisition protocol was chosen and acquired at two field strengths at two different centers. Eight b‐values were sampled per acquisition: b = 0 s/mm/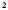 and seven nonzero b‐values b = [10, 50, 80, 200, 400, 600, 1000] s/mm/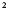, with all b>0 s/mm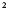 volumes acquired in three orthogonal diffusion directions, yielding 22 diffusion‐weighted volumes in total. Sequence parameters varied by field strength. At 0.55T: TR = 7200 ms, TE = 129 ms, TA = 2.43 min, FOV = 400 × 400 mm/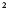, echo spacing = 0.97ms, no fat saturation, phase oversampling = 0%, slice distance factor = 0%, resolution = 3 × 3 × 3 mms/mm/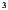, partial Fourier = 6/8 and 36 total slices. At 1.5T: TR = 4500 ms, TE = 88 ms, TA = 1.54 min, FOV = 300 × 300 mm^2^, echo spacing = 0.81ms, fat saturation = weak, phase oversampling = 10%, slice distance factor = 20%, resolution = 1.7 × 1.7 × 4 mm^3^, grappa = 2, partial Fourier = 6/8 and 30 total slices.

#### 2.1.2 Step 2: Fetal Brain Segmentation

Automated fetal brain segmentation is performed in real time on each acquired volume using a 3D nnU‐Net [15] trained in‐house, building on a previously published model (V1) [14] trained across 2690 images with a broad range of field strengths, vendors, and fetal imaging contrasts. While V1 demonstrated strong performance on standard acquisitions, generalization to cases with moderate to severe brain pathology and motion‐corrupted diffusion volumes remained limited. An updated network (V2) was therefore developed to improve robustness and generalisability across the wider range of acquisition settings evaluated in this work.

**FIGURE 2:**
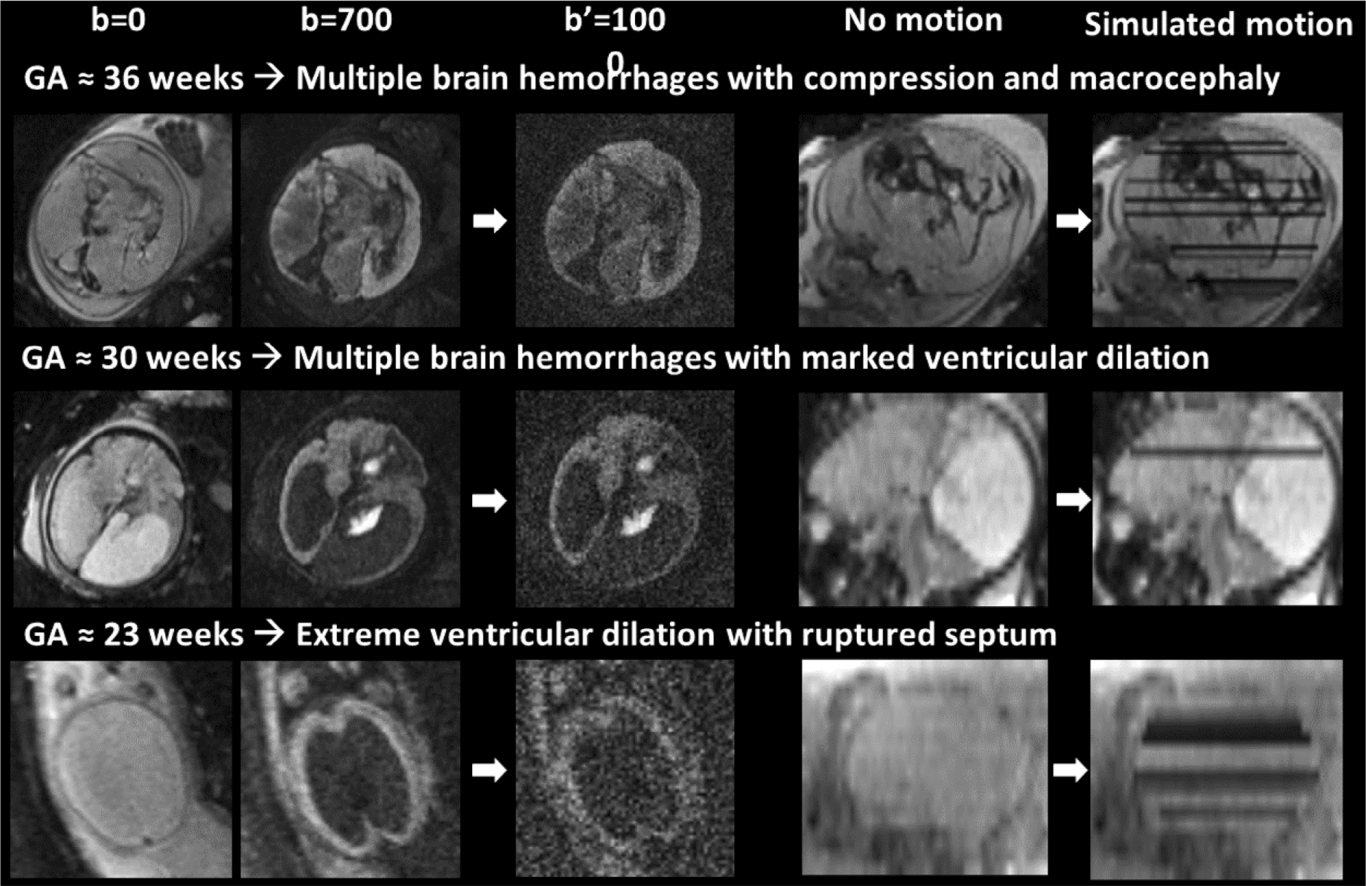
Segmentation strategy. Examples from the cohort focusing on fetal brain pathologies and corresponding augmentations. The first two columns show acquired axial fetal brain Diffusion‐weighted MR slices at b = 0 and b = 700 s/mm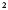. The third column presents simulated b = 1000 s/mm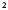 slices derived from the latter. The fourth and fifth columns show a coronal view, obtained by reformatting the stack of acquired slices at b = 0 s/mm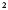 and the corresponding simulated image with motion‐related realistic artifacts.

V2 was obtained by fine‐tuning the V1 checkpoint for an additional 1000 epochs on an augmented dataset incorporating 60 anonymized clinical fetal dMRI examinations acquired at 1.5T (Ethics 24‐249‐Br), each containing a range of brain pathologies (Figure 2). As these clinical acquisitions followed a standard two b‐value protocol (b = 0 s/mm^2^ and b = 700 s/mm^2^ in three orthogonal directions), two augmentation strategies were applied for further generalization.

First, a synthetic b = 1000 s/mm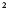 volume was generated from the available b = 700 s/mm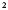 directions using a mono‐exponential signal attenuation model with a representative fetal ADC of 1.2 × 10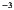 mm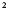/s, followed by Rician noise addition scaled to reflect the corresponding SNR reduction. This resulted in five motion‐free volumes per case: b = 0 s/mm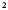, three orthogonal b = 700 s/mm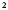 directions and the simulated b = 1000 s/mm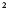 volume. Second, each of these five volumes was independently augmented with synthetic motion corruption by randomly attenuating between 10% and 50% of the slices containing brain tissue, with per‐slice intensity reduction factors drawn uniformly from [0.10, 0.65] of the original signal in line with analyzed retrospective data, producing five additional motion‐corrupted volumes per case. The augmented training set comprised a total of 598 additional volumes (59 cases × 10 volumes + 1 case × 8 volumes) combined with the original V1 training data for fine‐tuning. The network architecture was otherwise unchanged, with nnU‐Net preprocessing parameters updated automatically to reflect the expanded dataset. Representative examples of the acquired pathological cases alongside their corresponding augmented volumes are shown in Figure 2.

#### 2.1.3 Step 3: Artifact Detection

Artifact detection is performed in real time on each acquired volume using two complementary metrics: intra‐volume blackout slice detection and segmentation‐based brain volume change. Together, these determine whether a volume requires reacquisition and inform the prioritization strategy described in the following step. For blackout slice detection, the mean signal intensity within the brain mask is computed per slice and compared to the mean intensity across the entire brain region, with slices classified as motion‐corrupted if:

(1) $${Ī}_{\text{slice}}<(1-T)\cdot {Ī}_{\text{volume}}$$

with T = 0.35 applied throughout this work as determined by retrospective validation. The second metric monitors changes in the segmented brain volume (mm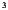) relative to that of the reference b = 0 s/mm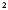 volume: severe intra‐volume motion can cause the segmentation to be spatially truncated, producing an apparent reduction in brain volume that serves as a surrogate indicator of a motion‐corrupted acquisition. A b‐value‐dependent threshold is applied to account for the natural signal attenuation associated with diffusion weighting.

#### 2.1.4 Step 4: Reacquisition Prioritization Strategy

Following artifact detection, each flagged volume is assigned to one of two reacquisition strategies: whole‐volume repetition or slice‐level repetition. This hybrid approach is the key methodological innovation of the present work, enabling targeted recovery of individual corrupted slices rather than discarding and repeating an entire diffusion‐weighted volume.

Volumes containing six or more corrupted slices, or those where segmentation‐based brain volume change exceeds the b‐value‐dependent threshold, are flagged for whole‐volume repetition. The six‐slice threshold was chosen empirically: given the small size of the fetal brain relative to the full imaging slab, six corrupted slices typically represent corruption of more than half of the brain‐intersecting slices, at which point the overhead of slice‐selective planning outweighs its benefit and a clean whole‐volume repeat is more efficient. The scanner offers the possibility to define diffusion weightings and directions to be acquired through a text file. In case of partial reacquisition, such a file is generated, containing the original diffusion gradient directions for each corrupted dynamic, preceded by a b = 0 s/mm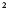 reference, and transferred to the scanner for reacquisition.

For volumes with fewer than six corrupted slices, a mixed diffusion‐preparation plan is constructed that assigns an independent diffusion gradient direction to each of the slice positions within a single TR reacquisition block. The plan is constructed in three sequential steps:

**Step 4a‐Corrupted slice assignment:** Each corrupted slice is assigned the diffusion preparation of its originating dynamic. When the same slice position is corrupted across multiple volumes, the lowest b‐value is prioritized, as these acquisitions retain higher SNR and contribute disproportionately more to downstream diffusion parameter estimation.

**Step 4b‐Neighborhood inclusion:** The immediately adjacent slices (±1 position) of each corrupted slice are added to the plan, inheriting the b‐value of their corrupted neighbor. Where a neighboring position falls between two corrupted slices with different b‐values, the lower b‐value is assigned.

**Step 4c‐Remaining slice assignment:** Slice positions that do not intersect the brain mask are filled with b = 0 s/mm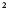 ensuring the total slice count remains fixed at 36 and preserving the acquisition duration and sequence parameters of the original protocol.

#### 2.1.5 Step 5: Hybrid Reacquisition

All real‐time processing steps following the initial acquisition‐brain segmentation, artifact detection, and reacquisition prioritization‐are executed automatically on a dedicated GPU workstation integrated with the scanner via the Siemens FIRE framework [16]. The resulting DVS files are transferred back to the scanner automatically, allowing reacquisition immediately upon completion of the analysis.

Two reacquisition sequences are run sequentially. The first sequence handles slice‐level repetition (Step 5a) and represents the key technical contribution of this work. In a conventional DW‐SE‐EPI sequence, all slices within a volume share the same diffusion preparation‐a single b‐value and gradient direction applied homogeneously across the entire slab. The original sequence was modified to break with this constraint, enabling individual slices within a single acquisition block to adopt distinct b‐values and gradient directions. This fundamentally alters the structure of the imaging sequence, pairing each slice position with an independently specified diffusion preparation rather than a shared one. Conceptually, this builds on flexible diffusion‐relaxometry frameworks that allow per‐slice preparation variation [17], but is significantly extended here to support freely specified slice‐preparation tables generated from external real‐time analysis. At scan time, the sequence reads the slice‐level DVS file directly, assigning one gradient vector per slice position. The resulting mixed diffusion‐preparation volume is acquired in a single block of single TR duration (in the prospective experiments shown below, for example, TA = 14s for 0.55T and TA = 31s for 1.5T), with all other sequence parameters identical to the original acquisition, after which the reacquired slices are available for retrospective replacement of their corrupted counterparts in the original dataset. The second sequence handles whole‐volume repetition (Step 5b): a standard diffusion sequence reads the whole‐volume DVS file, which specifies one gradient vector per dynamic, and repeats each flagged volume in its entirety. This sequence runs only if one or more volumes have been flagged for whole‐volume repetition; otherwise it is skipped.

The slice‐level diffusion preparation capability of the modified sequence was validated in a controlled phantom experiment on the 1.5T system, where the use of a static phantom vendor‐supplied, doped with 3,75g NiSo4 × 6H2O, 5g NaCl allowed a manually defined reacquisition plan to be executed and evaluated in isolation from the automated detection and prioritization steps, confirming the ability to assign distinct b‐values and gradient directions to individual slice positions within a single acquisition block. Validation was performed by calculating the ADC of the phantom with single‐encoding volumes and matching the expected decay of hybrid volumes over different b‐values with mono‐exponential decay.

### 2.2 Experiments

#### 2.2.1 Retrospective Datasets

Three retrospective datasets were used for validation, covering a range of field strengths, acquisition protocols, and clinical presentations.

**0.55T Cohort:** Twenty subjects were drawn from three studies acquired on the same 0.55T Siemens Magnetom Free. Max scanner under the same protocol: MEERKAT (Ethics REC 21/LO/0742), MiBirth (Ethics REC 23/LO/0685), and PRESTO (REC 21/SS/0082). As all three studies share identical acquisition parameters (22 preparations: b = 0 s/mm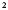 and b = [10, 50, 80, 200, 400, 600, 1000] s/mm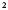 in three orthogonal directions), they were combined into a single cohort for analysis. Data were acquired in axial orientation aligned to the fetal brain. All subjects were healthy volunteers at the time of recruitment, and 20 were selected randomly from the combined whole datasets.

**1.5T Clinical Cohort:** Twenty anonymized clinically referred fetal MRI cases (Ethics 24‐249‐Br) acquired at a 1.5T Sola Siemens scanner were selected from the same clinical dataset used for V2 network fine‐tuning, with no overlap between training and validation cases. Cases were chosen upon visual inspection to cover a range of moderate to severe CNS pathologies. Data were acquired in axial or coronal orientation aligned to the fetal brain, comprising four preparations: b = 0 s/mm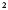 and b = 700 s/mm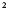 in three orthogonal directions.

**1.5T Cohort:** Twenty randomly selected subjects were drawn from the CARP dataset [18, 19] (Ethics REC 08/LO/1958), acquired at a 1.5T Philips Ingenia scanner in a mixed cohort of healthy subjects and subjects with pre‐eclampsia. Data were acquired in coronal orientation with a whole‐uterus field of view, comprising 13 preparations: b = 0 s/mm/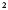 and b = 375 s/mm/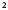 and b = 750 s/mm/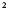 in six orthogonal directions.

#### 2.2.2 Retrospective Evaluation

Retrospective validation was performed on 60 cases drawn equally from the three cohorts described above (20 per cohort). For each case, three volumes were selected: the b = 0 s/mm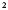 volume and two additional volumes chosen to provide variability across the b‐value range and motion corruption levels. For the 0.55T and nonclinical 1.5T cohorts, the two non‐b = 0 volumes were selected randomly from those containing motion‐induced corruption. For the 1.5T Clinical cohort, the two non‐b = 0 volumes corresponded to the first two orthogonal directions of b = 700 s/mm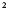; as these cases were acquired clinically and are largely free of motion artifacts, volume selection was fixed rather than corruption‐driven. This yielded a total of 180 volumes for evaluation.

**Segmentation Validation:** Manual reference segmentations of the fetal brain were produced by two experienced fetal MRI researchers, each annotating 90 of the 180 images, with both observers blinded to the automatic segmentation results. Automatic segmentations were generated for all 180 volumes using both V1 and V2, and performance was quantified using the Dice similarity coefficient (DSC) computed against the available manual reference for each volume. DSC was analyzed separately for the b = 0 s/mm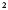 volume and the higher b‐value volumes to assess the impact of diffusion weighting and motion corruption on segmentation performance, and results are reported per cohort to reflect the different acquisition challenges presented by each dataset.

**Blackout Slice Detection Validation:** The same two observers independently and visually identified corrupted slices within each of the 180 volumes, blinded to the automatic detection results, providing a manual reference for validation. Automatic blackout detection was performed across four intensity drop thresholds (T = 0.25,0.35,0.45,0.55) to characterize the sensitivity‐specificity trade‐off and demonstrate that the chosen operating threshold was selected based on empirical evidence rather than arbitrary choice. Detection performance was quantified using sensitivity, specificity, and F1 score computed against the manual annotations, and reported overall and separately for the 0.55T and 1.5T cohorts. The 1.5T clinical cohort was excluded from this analysis as the absence of motion artifacts in these cases precludes meaningful evaluation of blackout detection performance.

#### 2.2.3 Prospective Datasets and Evaluation

The pipeline was evaluated prospectively first in phantom experiments to further confirm the slice‐level diffusion preparation capability of the modified sequence in isolation (Figure 6). Then, the fully operational pipeline was deployed in 14 in‐vivo fetal cases at two sites, 6 healthy subjects acquired at 0.55T (Siemens Magnetom Free.Max) and 8 subjects with different pathologies (2 cases with ventriculomegaly, 1 case with complex CNS pathologies, 1 case with an upper lip tumour, 2 cases with placental pathologies, one of them with anhydramnios, 1 case with hydronephrosis and 1 case with maxilo‐nasal dysplasia) acquired at 1.5 T (Siemens Magnetom Sola) at a different site, following the protocols described in Step 1. At both field strengths and in all cases, the complete real‐time analysis (Steps 2–4), brain segmentation, and artifact detection, reacquisition prioritization was performed at the scanner fully automatically. In addition, the reacquisition sequence execution (Step 5) was executed in all cases at 0.55T and 6 cases at 1.5T, omitted in 2 cases due to clinical workflow constraints. Results for Steps 2–4 are therefore reported for all 14 prospective subjects, while end‐to‐end pipeline evaluation including reacquisition is reported for the 6 0.55T cases and 6 1.5T cases where the full workflow was completed. Segmentation quality was assessed through visual inspection by an experienced observer, who rated each automatically generated brain segmentation as acceptable (1), minor modification (2), or major modification (3). Blackout slice detection was evaluated by the same observers through visual inspection of the acquired volumes, recording agreement or disagreement with the pipeline's reacquisition decisions.

## 3 Results

### 3.1 Retrospective Evaluation

#### 3.1.1 Brain Segmentation

**FIGURE 3:**
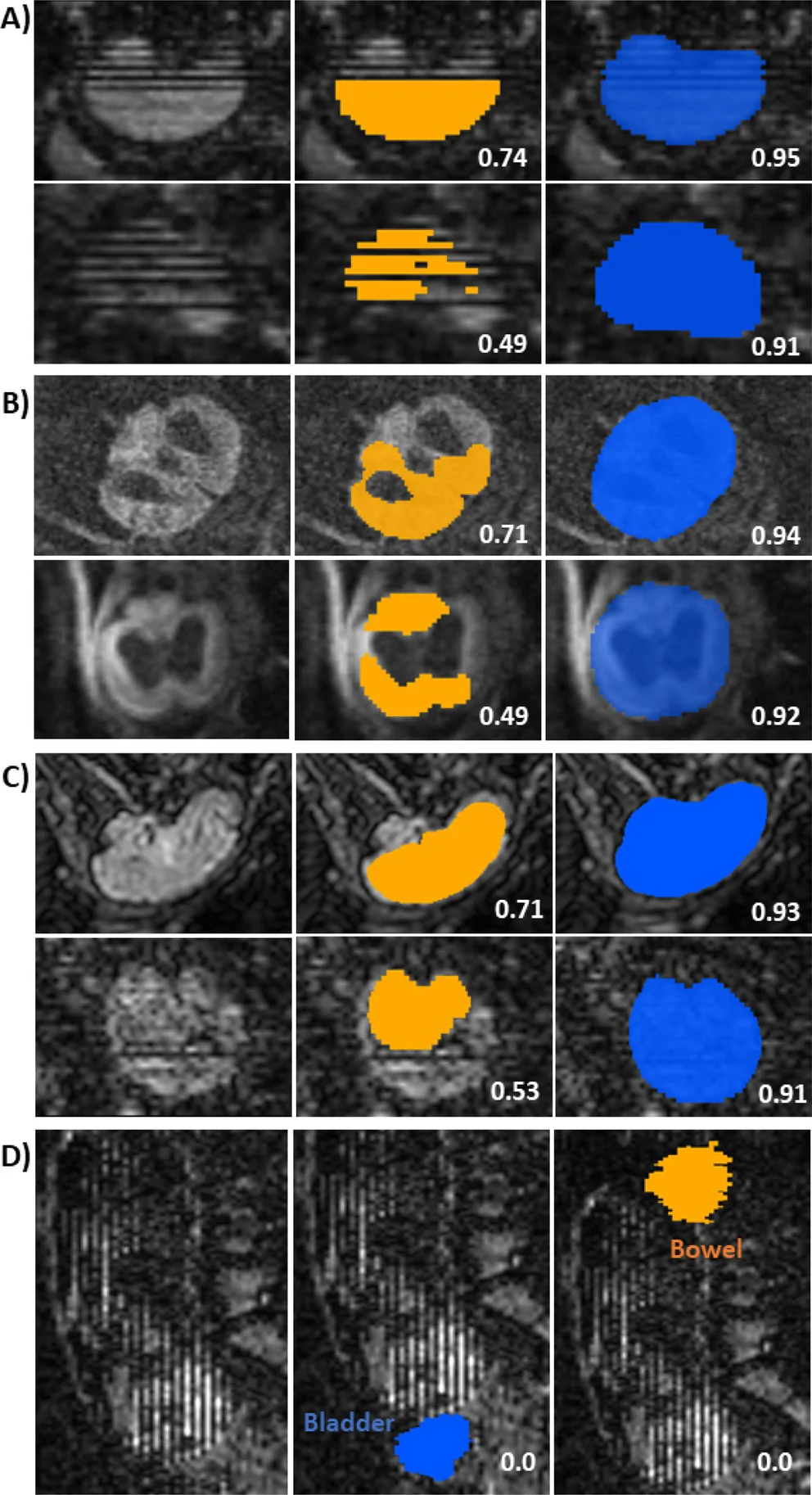
Representative fetal brain segmentation examples. Results are depicted for the original network (V1, orange) and the proposed novel version (V2, blue) with corresponding Dice scores. For each group, a case of moderate improvement (top, defined as Dice improvement of at least 0.2) and large improvement (bottom, defined as Dice improvement of at least 0.35) is shown. **(A)** 0.55 T cohort. **(B)** 1.5 T Clinical cohort. **(C)** 1.5 T cohort. **(D)** illustrates a failure case (Dice Score = 0 for both network versions).

**FIGURE 4:**
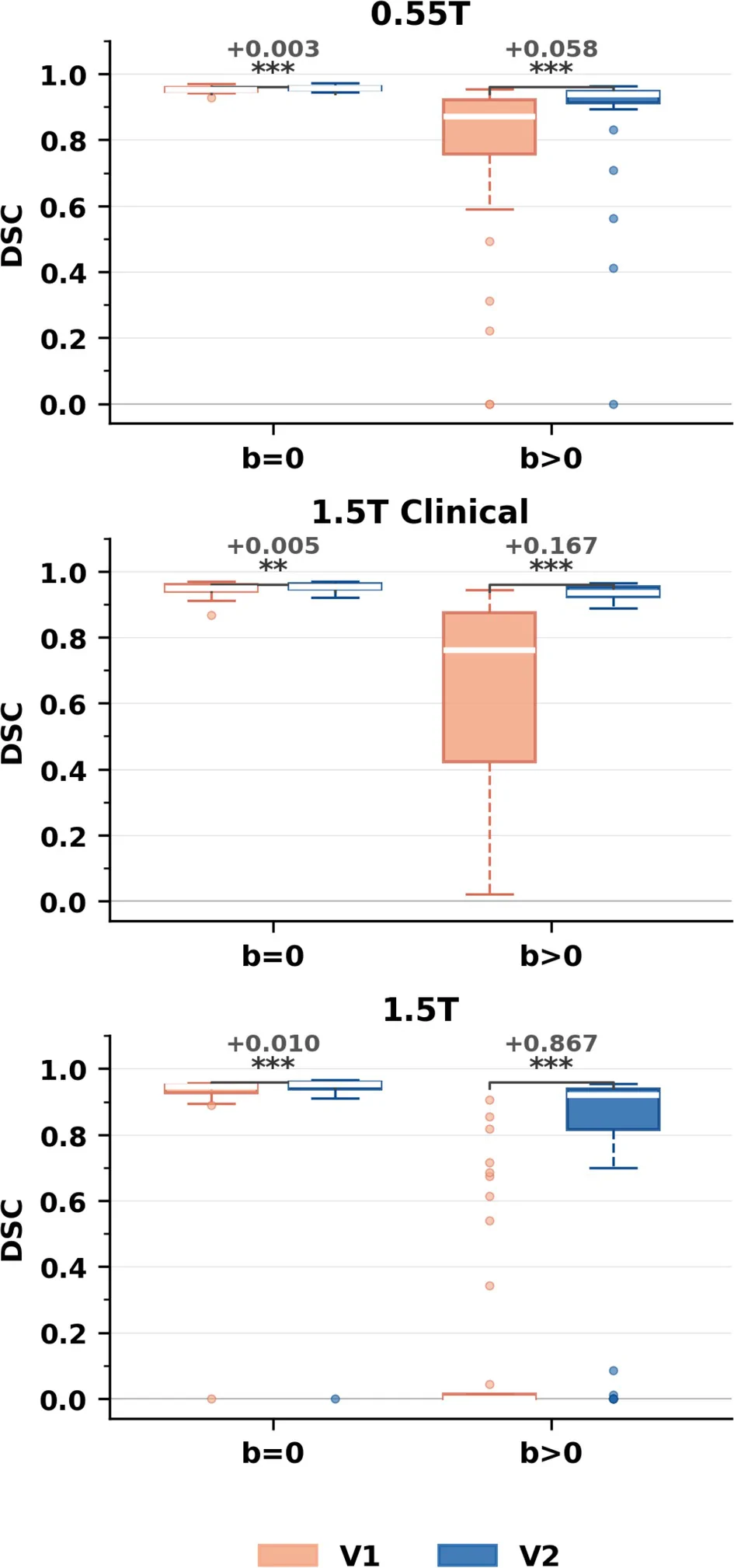
Evaluation of the fetal brain segmentation performance. Quantitative Dice Scores are shown for the original and proposed network versions (V1 and V2) across three cohorts at b = 0 s/mm/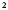 and b>0 s/mm/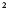. The boxes show interquartile ranges, white lines indicate the median, and diamonds indicate the mean. Paired median gain (V2 – V1) and significance are shown above each pair (Wilcoxon signed‐rank: ** p<0.01, *** p<0.001).

DSC was computed against manual reference segmentations, reported separately for the b = 0 s/mm/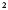 reference volume and higher b‐value volumes (b>0 s/mm/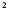) per cohort (Figures 3, 4).

At b = 0 s/mm/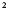, both versions performed consistently across all cohorts. Mean DSC for V1/V2 was 0.952±0.009/0.956±0.007 (0.55 T, median gain +0.003, p<0.001), 0.946±0.022/0.953±0.022 (1.5 T Clinical, gain +0.005, p=0.001), and 0.891±0.205/0.902±0.207 (1.5 T, gain +0.010, p=0.001); one case in the latter cohort yielded DSC = 0 for both versions.

At b>0 s/mm/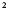, V2 substantially outperformed V1 across all cohorts. Mean DSC for V1/V2 was 0.774±0.241/0.882±0.177 (0.55 T, median gain +0.058, p<0.001), 0.636±0.282/0.935±0.018 (1.5 T Clinical, gain +0.167, p<0.001; V2 exceeded V1 in all 40 volumes, no V2 segmentation fell below DSC = 0.888), and 0.155±0.295/0.769±0.322 (1.5 T, gain +0.867, p<0.001; V1 produced DSC = 0 in 27 of 40 volumes compared to 4 of 40 for V2).

#### 3.1.2 Motion Artifact Detection

**FIGURE 5:**
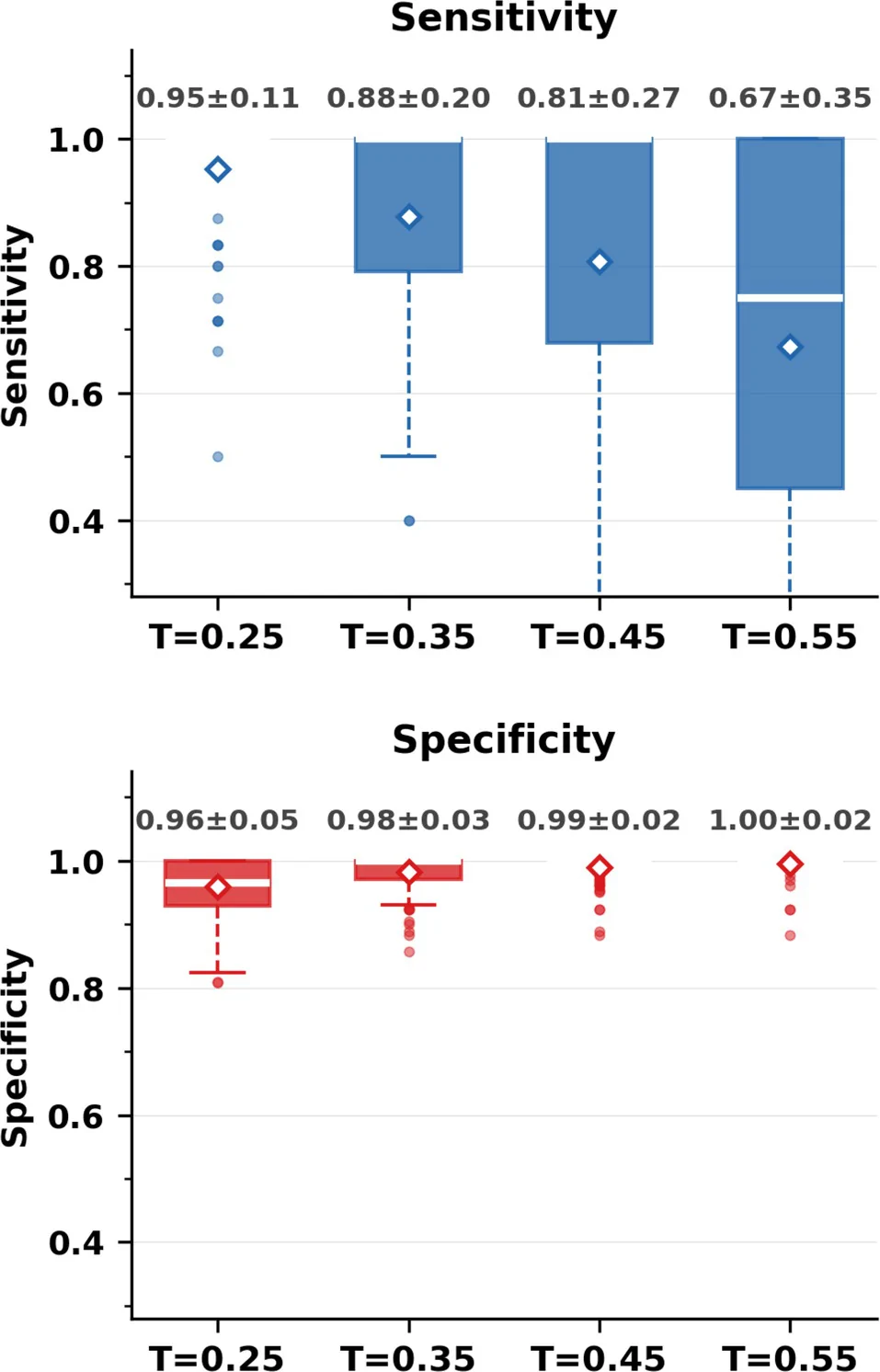
Artifact (Blackout slice) detection performance evaluation. Results across four intensity thresholds (T = 0.25, 0.35, 0.45, 0.55) are shown, compared with manual annotations across the retrospective cases from the 0.55 T and 1.5 T combined, 1.5T Clinical excluded cohorts. The boxes show interquartile ranges, white lines indicate the median, and diamonds indicate mean (mean±sd shown above each box).

Blackout slice detection performance across all four thresholds is summarized in Figure 5 and Table 1.

**TABLE 1:** Evaluation of the artifact (blackout slice) detection performance and of the applied threshold. Results are reported across field strengths (0.55 T and 1.5 T combined) and per cohort. Values are mean ± standard deviation. The best threshold per cohort is shown in bold.

Cohort	Threshold	Sensitivity	Specificity	F1
Overall	T = 0.25	0.953±0.107	0.960±0.048	0.746±0.340
	T **= 0.35**	0.878±0.207	0.982±0.034	0.812±0.290
	T = 0.45	0.808±0.268	0.989±0.025	0.799±0.285
	T = 0.55	0.673±0.352	0.995±0.019	0.728±0.328
0.55 T	T = 0.25	0.972±0.074	0.970±0.035	0.880±0.182
	T **= 0.35**	0.904±0.200	0.986±0.029	0.903±0.191
	T = 0.45	0.862±0.237	0.992±0.024	0.886±0.208
	T = 0.55	0.729±0.337	0.998±0.012	0.781±0.318
1.5 T	T = 0.25	0.925±0.138	0.949±0.057	0.604±0.407
	T **= 0.35**	0.840±0.213	0.977±0.039	0.705±0.349
	T = 0.45	0.731±0.294	0.987±0.026	0.688±0.331
	T = 0.55	0.593±0.363	0.992±0.024	0.652±0.332

A clear sensitivity‐specificity trade‐off was observed with increasing threshold. T = 0.35 achieved the best overall balance, yielding mean sensitivity of 0.878±0.207, specificity of 0.982±0.034, and the highest F1 of 0.812±0.290, significantly outperforming T=0.45 (p=0.042, Wilcoxon signed‐rank). At T=0.35, perfect detection was achieved in 61% and 42% of 0.55 T and 1.5 T volumes, respectively. F1 was significantly higher at 0.55T than at 1.5T (0.903±0.191 vs. 0.705±0.349, Mann‐Whitney p=0.004), while sensitivity (0.904±0.200 vs. 0.840±0.213, p=0.234) and specificity (0.986±0.029 vs. 0.977±0.039, p=0.194) did not differ significantly between cohorts.

### 3.2 Prospective Evaluation

**FIGURE 6:**
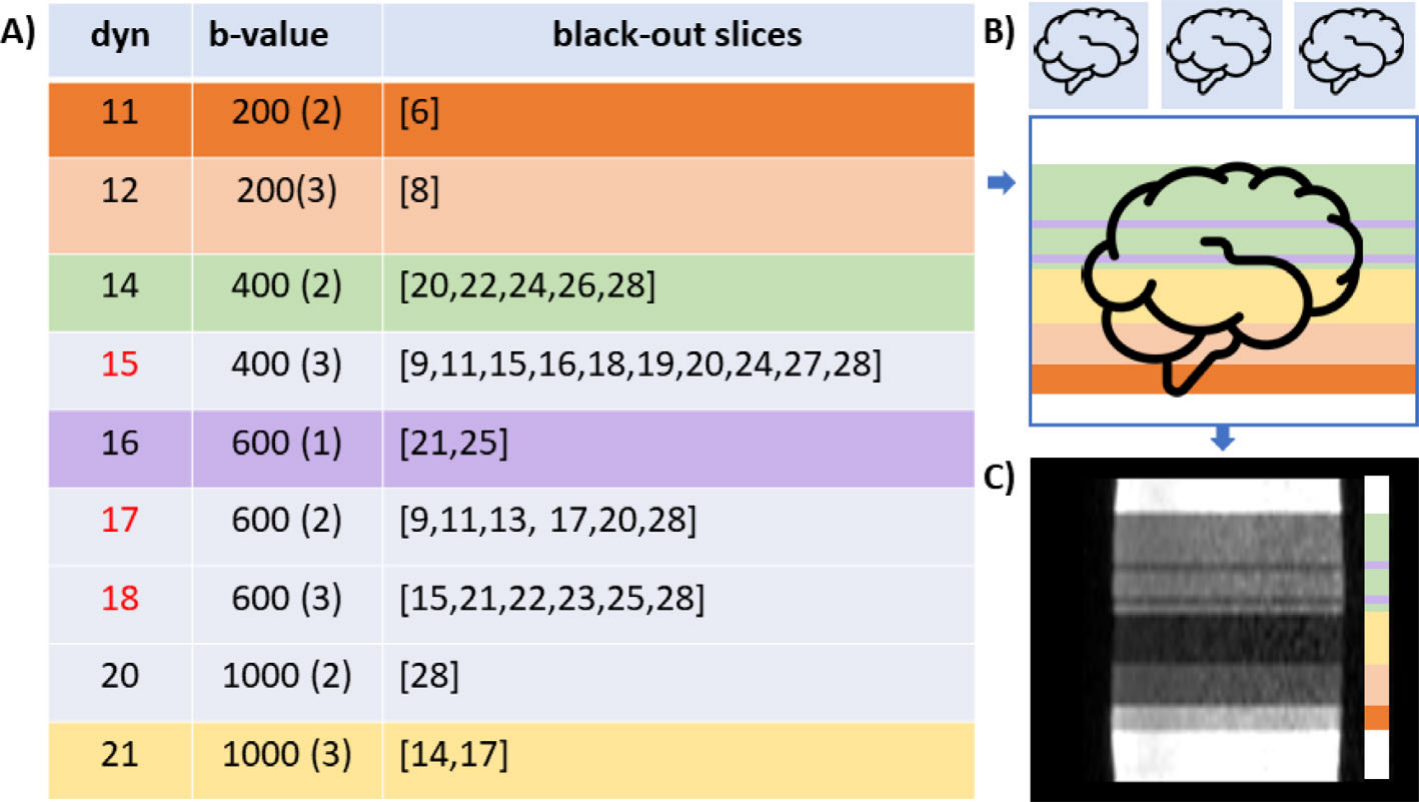
Phantom demonstration. The prospectively acquired mixed volumes with varying slice‐level diffusion preparation are shown. **(A)** Blackout slices per volume: volumes with ≥6 corrupted slices are flagged for whole‐volume repetition (light blue) and remaining volumes are assigned to the slice‐level plan, each in a distinct color varying by b‐value. **(B)** Resulting mixed diffusion‐preparation reacquisition plan. **(C)** Acquired phantom volume showing the prescribed slice‐level diffusion pattern.

Prospective evaluation at 1.5T comprised 8 cases and 176 volumes. Segmentation scored 1 in 158 of 176 volumes (89.8%) and 2 in the remaining 18 (10.2%), with no unacceptable segmentations. Manual inspection identified blackout slices in 10 of 176 volumes across 3 cases; automatic detection correctly identified 8, missed 2 (single slices at the brain border), and generated 6 false positives each involving a single slice. Of 6 cases with full pipeline execution, 2 were motion‐free, 3 had corrupted slices successfully reacquired, and 1 was triggered by false positive detections. A phantom experiment further confirmed slice‐level diffusion preparation on the 1.5 T system (Figure 6). The results were consistently available in <60sec after the acquisition of this individual sequence.

Prospective evaluation at 0.55 T comprised 6 cases and 132 volumes. Segmentation scored 1 in 123 of 132 volumes (93.2%), 2 in 4 volumes (3.0%), and 3 in 5 volumes (3.8%); one case contributed 4 unacceptable segmentations predominantly at higher b‐value volumes due to incomplete brain delineation, resulting in complete reacquisition of these concerned volumes. Manual inspection identified blackout slices in 17 of 132 volumes across 4 cases, with 79 annotated blackout slices in total. Automatic detection flagged 17 volumes with 60 detected slices: 11 volumes were perfectly detected, 1 was missed, and 5 were partially detected, predominantly at the brain mask boundary or in heavily corrupted volumes. One additional volume was flagged without a corresponding manual annotation.

**FIGURE 7:**
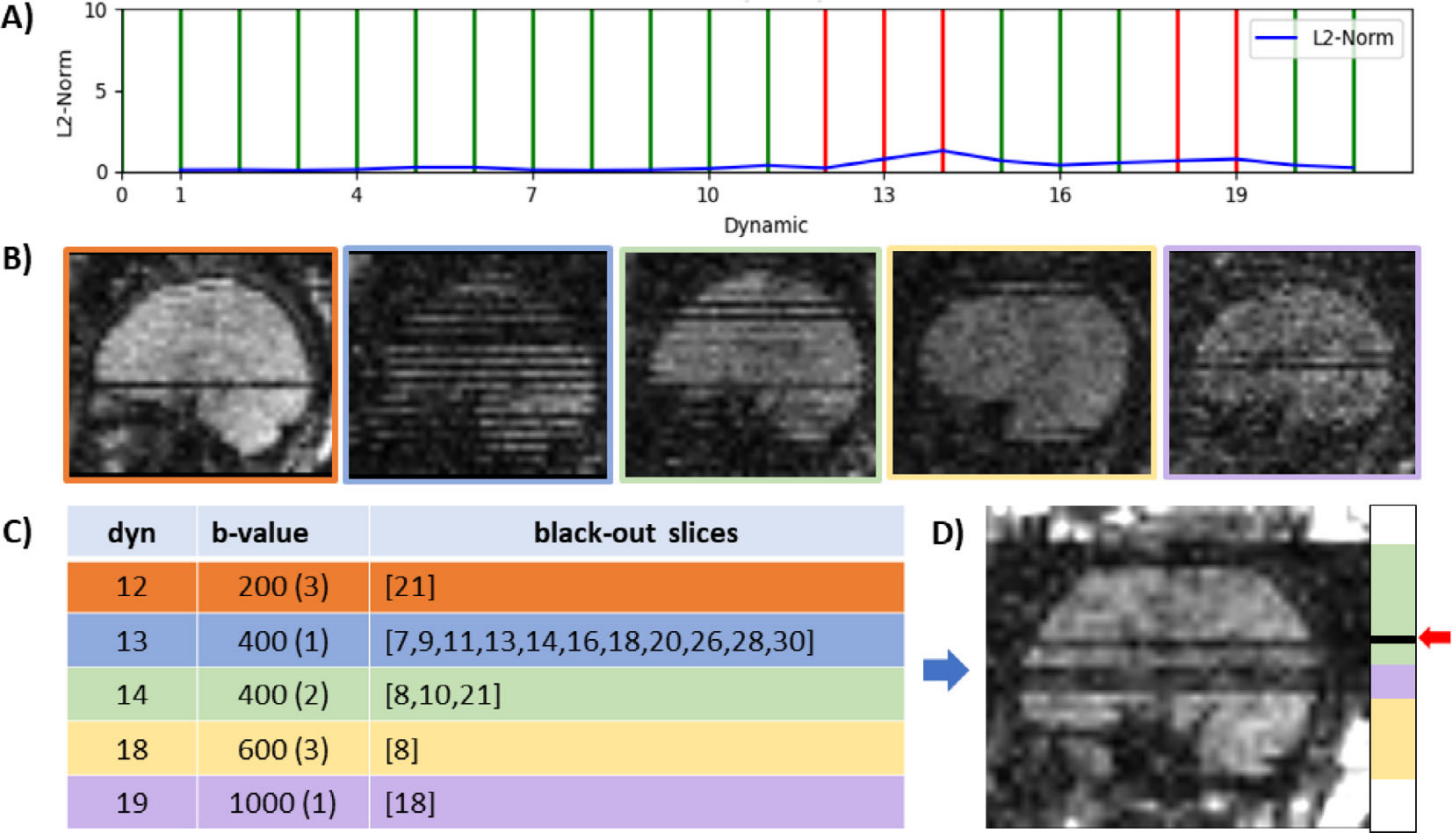
Representative end‐to‐end pipeline example from a prospective 0.55T acquisition. **(A)** Real‐time quality analysis: green lines indicate motion‐free volumes, red lines indicate volumes flagged for reacquisition. **(B)** Reformatted coronal slices from the five flagged volumes, color‐coded by diffusion preparation. **(C)** Detected blackout slices per flagged volume; dynamic 13 (blue) was flagged for whole‐volume repetition (≥6 corrupted slices). **(D)** Resulting mixed diffusion‐preparation reacquisition volume; the red arrow indicates a residual dropout in a neighboring buffer slice.

**FIGURE 8:**
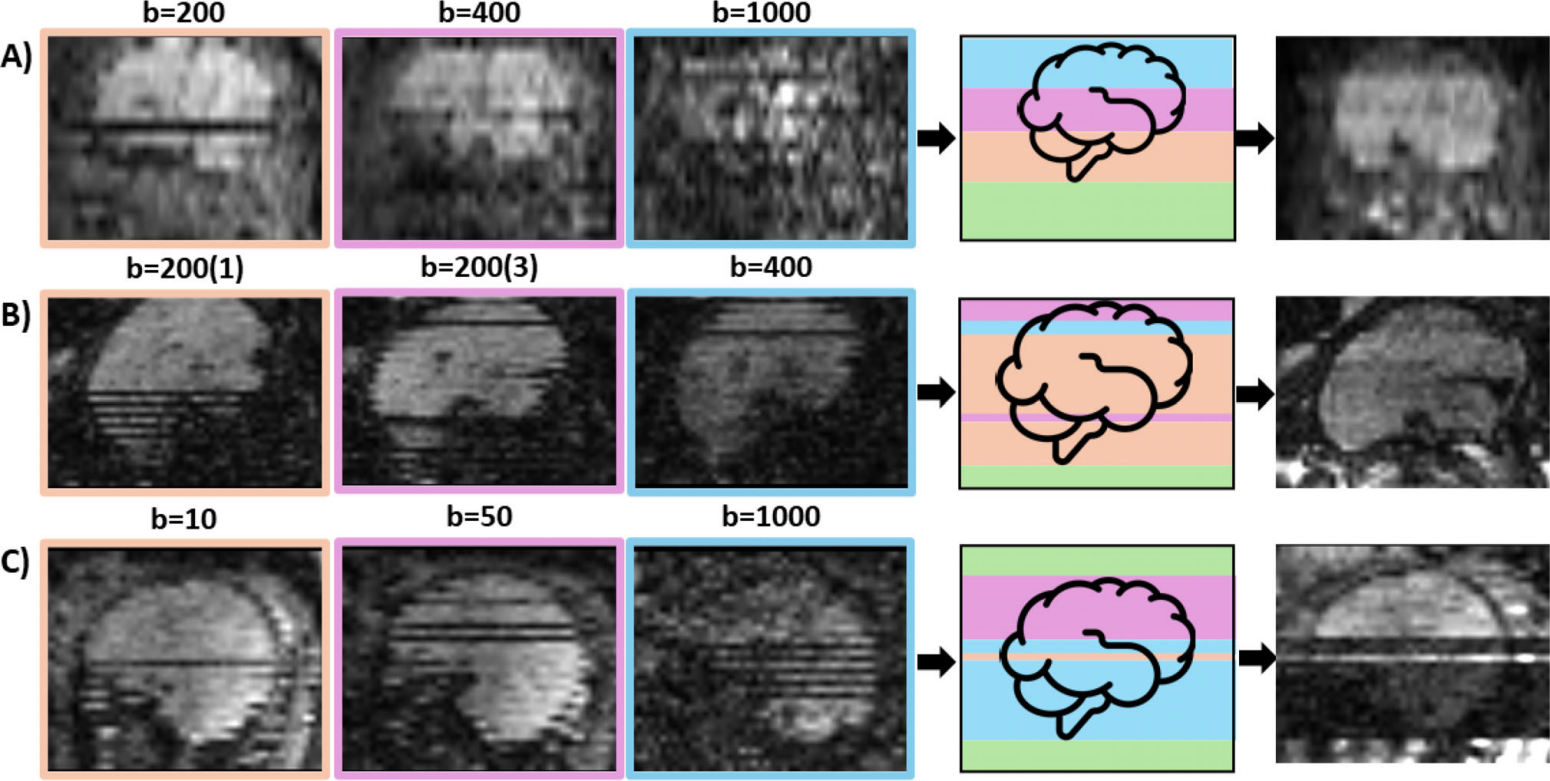
Prospective acquired cases with the proposed framework. Examples of slice‐level reacquisition plans from prospective cases. For each case, the first three columns show representative corrupted volumes selected for slice‐level reacquisition, the fourth column shows the resulting mixed diffusion‐preparation plan, and the fifth column shows the acquired reacquisition volume. **(A)** 1.5T case with individual black out slices in all b‐values. **(B)** 0.55T case with corruption in consecutive diffusion preparations. **(C)** 0.55T case with corruption spanning a wide b‐value range.

A representative end‐to‐end pipeline example is shown in Figure 7. The residual dropout (red arrow, panel D) corresponds to a neighboring buffer slice rather than a directly corrupted slice, confirming successful target slice recovery. Further examples across both field strengths are shown in Figure 8. The 1.5T case (Figure 8A) demonstrates pipeline operation in a clinical setting; the two 0.55T cases illustrate adaptation to different corruption patterns‐consecutive preparations with similar b‐values (Figure 8B) and corruption spanning the full b‐value range (Figure 8C). Volumes shown represent a subset of those flagged‐only those assigned to the slice‐level plan are depicted, with volumes exceeding the whole‐volume threshold handled separately.

**FIGURE 9:**
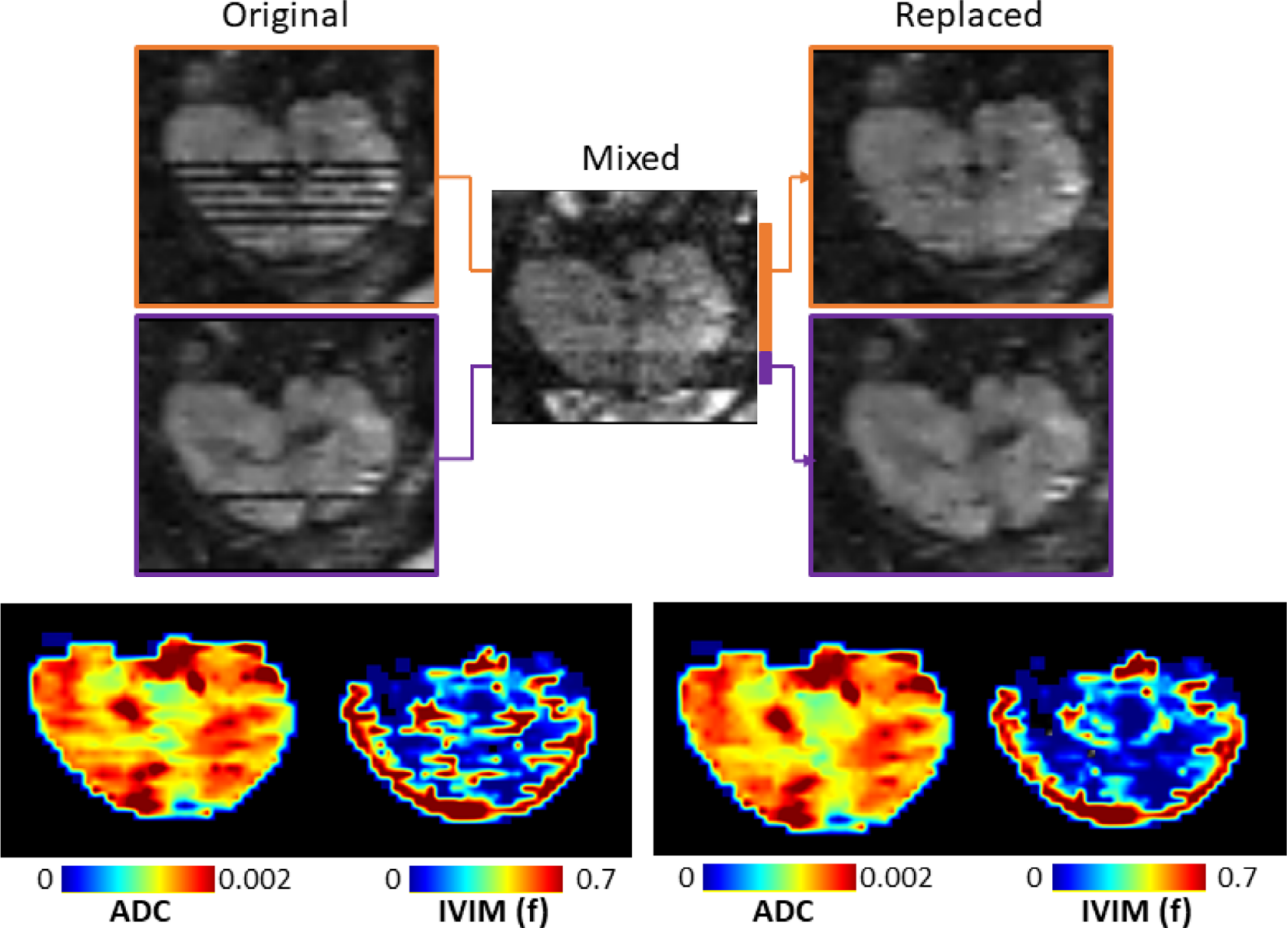
Slice‐level reacquisition analysis in a representative prospective 0.55T case. Left: Corrupted volumes from the original acquisition, showing motion‐induced blackout artifacts, with the corresponding ADC and IVIM perfusion fraction maps; Middle: the single mixed‐preparation reacquisition block acquired by the proposed pipeline, containing reacquired slices for both corrupted volumes; Right: same volumes after slice‐level replacement, with the corresponding ADC and IVIM f maps, showing reduced motion artifacts and more spatially homogenous parameter estimates.

The results of the derived diffusion quantification in Figure 9 illustrate the effect of replacing artifacted slices with the automatically reacquired slices in a mixed volume by showing reduction in slice artifacts and more consistent ADC and perfusion fraction maps. The increased efficiency in terms of reduced time required for the reacquisition compared to both conventional and the previously proposed full‐volume reacquisition is shown in supporting Table S1, with a mean reduction of 50.1% and 94.1%, respectively.

## 4 Discussion

### 4.1 Summary

This work presents a complete framework for real‐time fetal diffusion MRI, introducing two key contributions: a segmentation network with robustness to brain pathology, reduced SNR and motion corruption, and an acquisition sequence breaking with the conventional one‐volume one‐preparation paradigm by enabling slice‐level diffusion preparation within a single reacquisition block. Together, these advances shift the reacquisition strategy from volume‐level repetition to targeted slice‐level repetition, hence substantially reducing scan inefficiency while preserving the diffusion information required for downstream analysis. Retrospective validation across 60 cases and three cohorts demonstrated consistent segmentation improvement with V2, with the largest gains observed in the most challenging acquisition settings. Prospective evaluation confirmed real‐time feasibility across both field strengths.

### 4.2 Segmentation Network

The improvement from detection network V1 to V2 was most pronounced at higher b‐values and in cases of severe pathologies, where motion‐induced signal loss or pathology‐related anatomical variations fundamentally alter the appearance of the fetal brain. V1 often segmented the brain correctly in low‐corruption volumes but excluded slices affected by blackout artifacts from the brain mask, limiting the reliability of downstream intensity‐based detection. V2 consistently maintained anatomical completeness across these slices, enabling accurate blackout identification and slice‐level reacquisition planning. In cases with severe pathologies involving large fluid spaces or ventricular dilatation, V2 delineated the brain more accurately than V1, preserving the mask integrity on which both detection metrics depend (see Figure 3). This has a compounding benefit: improved segmentation directly improves DSC and indirectly improves blackout detection, since both metrics are downstream of mask quality. In the prospective 0.55 T cases, partial detections were consistently associated with spatially truncated brain masks; in the same cases, segmentation failure triggered whole‐volume reacquisition via the volume change metric, demonstrating its value as a safety net when segmentation is challenged. Retaining both metrics therefore remains the most robust strategy.

### 4.3 Blackout Slice Detection

The retrospective validation confirmed a clear one‐directional sensitivity‐specificity trade‐off across the four evaluated thresholds, with T = 0.35 achieving the best F1 in both cohorts independently, supporting its use as a single operating threshold across field strengths and acquisition protocols. T = 0.35 reflects the asymmetric costs of false positives and false negatives: the former adds marginally to scan time, while the latter misses a genuinely corrupted slice. T = 0.35 maintains high sensitivity while keeping false positives low enough not to burden the clinical workflow.

The lower F1 at 1.5 T relative to 0.55 T was driven primarily by a higher false positive rate in the 1.5T cohort, consistent with the greater complexity of motion‐induced signal loss patterns in coronal whole‐uterus acquisitions. Sensitivity was comparable between cohorts, indicating the threshold generalizes well across field strengths and acquisition geometries. In the prospective 1.5T Clinical cases, very few blackout slices were detected overall‐fewer than expected from the clinical setting. This likely reflects the acquisition protocol used in this cohort, designed for visual clinical assessment rather than quantitative diffusion analysis, which may attenuate motion‐induced signal dropout and render blackouts more subtle. This has an important implication: the absence of detected blackouts in this cohort does not necessarily reflect true absence of data corruption, and the protocol is not ideal for quantitative diffusion metrics including ADC or IVIM mapping.

### 4.4 Comparison to the State of the Art

The vast majority of fetal diffusion motion frameworks operate on the acquired data after the exam is completed without allowing adaptations to the acquisition itself [5, 6, 7, 20], whereas the proposed approach allows real‐time reactive adaptation. On the acquisition side, compared to previous work changing diffusion preparations within the volume in a predefined way [17, 21], the current study allows dynamic changes per slice triggered by real‐time input. On the other side, the focus of the present study on the real‐time detection and reacquisition leads to it falling short compared to alternative approaches [5, 6, 20] regarding dedicated motion‐informed processing and quantification.

### 4.5 Strengths and Limitations

The primary strength of this work is the synergistic combination of a novel acquisition strategy allowing flexible slice‐level diffusion preparation changes and slice‐level AI‐based artifact detection. It thereby allows to demonstrate real‐time slice‐level diffusion reacquisition in fetal MRI‐to the best of our knowledge, the first such implementation‐within a clinically feasible time budget. The thorough retrospective evaluation across 60 cases spanning three cohorts, two field strengths, diverse acquisition protocols, healthy volunteers, and cases with significant CNS pathologies provides a robust characterization of both the segmentation network and the detection algorithm across a wide range of real‐world conditions. The prospective real‐time deployment in two sites with two different field strengths, including in clinical cases with pathologies, demonstrates practical feasibility beyond a research setting and provides despite the small cohort size, early evidence of clinical translation. The dual‐metric detection approach, combining intensity‐based blackout detection with segmentation‐based volume change, provides built‐in redundancy that maintains reliable reacquisition triggering even when individual components are challenged. Together, these elements carry the early promise to contribute to enabling more complete and efficient diffusion datasets than previously achievable in a real‐time fetal MRI setting.

However, there are also limitations. The pipeline assumes low bulk fetal motion and minimal head position change between acquisitions; severe intervening motion could misalign reacquired slices with their intended positions. Detection performance, while strong overall, did not reach perfect sensitivity and specificity in all cases, with occasional missed blackout slices and false positive triggers particularly in the challenging higher b‐values. While occasional false positive slices still maintain increased efficiency while avoiding irreplaceable data loss, segmentation failures and missed peripheral blackout slices contain the risk of such clinically relevant information. Segmentation masks and detected motion, including the extent, are graphically available on the connected computer to allow supervision and manual readjustment where necessary, could however be displayed more readily available in the future. The threshold of six slices for complete volume reacquisition might be suboptimal for younger GAs, where even fewer slices should trigger a reacquisition, calling for a volume‐dependent threshold in the future. Furthermore, the current implementation generates a single mixed‐preparation reacquisition volume, which may not recover all affected slices when corruption is distributed across many volumes simultaneously. In addition, motion corruption in training was introduced exclusively through synthetic augmentation, whose fidelity relative to real artifacts warrants further investigation. The two cohorts employed different acquisition protocols, limiting direct field strength comparison while demonstrating robustness across settings. Finally, while prospectively tested in a total of 14 cases, validation in a larger prospective cohort is key to fully understand the potential impact of the newly opened mixed‐volume prospective reacquisition of the diffusion volumes.

### 4.6 Future Work

Acquisitions with a large number of diffusion directions, such as HARDI [3, 7], represent an ideal next test case, where the ability to compose a single reacquisition volume from corrupted slices across multiple direction volumes has the potential to yield even greater efficiency gains. Reacquisitions with changing diffusion preparations and motion patterns across slices potentially result in different eddy current and distortion artifacts. While observed neither in the phantom nor the in‐vivo data during this study, future research could focus on this, potentially in combination with the previously proposed dual‐echo approach [20, 21].

On the analysis side, the paradigm shift from volume‐level to slice‐level reacquisition should be complemented by corresponding advances in reconstruction and modelling. Slice‐to‐volume registration approaches [5] are the natural complement to the slice‐level data produced by this pipeline, and diffusion model fitting strategies capable of handling per‐slice diffusion preparation variation will be needed to extract quantitative parameters from the resulting heterogeneous datasets. Further work will focus on obtaining reliable ADC and IVIM maps from prospectively corrected fetal dMRI data.

A broader clinical implication is worth highlighting: the pipeline's reacquisition strategy could potentially make quantitative diffusion MRI routinely achievable in clinical settings where it is currently considered out of reach. In standard clinical fetal MRI, protocols are optimized for visual assessment at the cost of quantitative suitability‐thick slices, slice overlap, and limited b‐value sampling prioritize image quality for the reporting radiologist. With the present approach, the radiologist retains motion‐free images from several acquisitions while the pipeline recovers the corrupted data needed for microstructural analysis within a single short reacquisition block, without requiring protocol modifications or significant scan time extension. This could in the future open a pathway to routine quantitative fetal brain characterization in clinical populations, including those with CNS pathologies where microstructural information is most clinically valuable. Integration with automatic fetal brain plane localization [22] will further strengthen the pipeline by re‐prescribing the reacquisition block in a fetal‐anatomy‐aligned frame regardless of intervening fetal motion, addressing temporal heterogeneity between the initial and reacquired slices at the source and ensuring consistency of both slice positions and diffusion gradient directions relative to the fetal anatomy. Finally, validation in a larger prospective cohort across a wider range of gestational ages, pathologies, and clinical centers will be essential to establish the generalizability and clinical impact of the approach.

## 5 Conclusion

This work demonstrates real‐time slice‐level diffusion reacquisition in fetal MRI, combining a robust segmentation network and artifact detection strategy with a modified sequence capable of assigning different diffusion preparations per slice. Retrospective and prospective validation‐albeit in a small cohort‐confirmed consistent performance across field strengths, acquisition protocols, and clinical presentations including severe brain pathology. By targeting only corrupted slices rather than repeating entire volumes, the framework carries the potential to substantially improve acquisition efficiency while preserving diffusion data quality, opening after validation in a larger cohort a pathway toward routine quantitative fetal brain characterization in both research and clinical settings.

## Funding

This work was partially supported by the Deutsche Forschungsgemeinschaft (Grant No. 502024488), a European Research Council Starting Grant EARTHWORM (Grant No. 101165242), Medical Research Council funding (Grant No. MR/X010007/1) and the Lower Saxony Ministry of Science and Culture (Grant No. ZN4257).

## Conflicts of Interest

Sarah McElroy is a Siemens Healthineers employee. Raphael Tomi‐Tricot is a Siemens Healthineers employee.

## Supporting information




**Table S1.** Acquisition cost comparison across the 14 prospective cases for three reacquisition strategies:conventional whole‐acquisition repetition, conventional whole‐volume repetition, and the proposed slice‐level reacquisition.

## Data Availability

The data that support the findings of this study are available on request from the corresponding author. The data are not publicly available due to privacy or ethical restrictions.
