# Supplementary material for: Real‐Time Targeted Slice Reacquisition for Motion‐Corrupted Fetal Diffusion MRI
Source: Magn Reson Med. 2026 Jul 10;96(5):2140–51. doi: 10.1002/mrm.70484 (PMC13527251; doi:10.1002/mrm.70484)
Supplement: Supplementary file 1 [file MRM-96-2140-s001.pdf]

Case	Volumes with corruption	Conventional — whole acquisition repeated	Conventional — whole volume repeated	Proposed	Time reduction vs. whole scan repetition	Time reduction vs. whole volume repetition
Case 1	0 volumes	—	—	—	0%	0%
Case 2	3 volumes	22 volumes	3 volumes	1 volume	95.5%	67%
Case 3	5 volumes	22 volumes	5 volumes	2 volumes	90.9%	60%
Case 4	5 volumes	22 volumes	5 volumes	3 volumes	86.4%	40%
Case 5	4 volumes	22 volumes	4 volumes	1 volume	95.5%	75%
Case 6	0 volumes	—	—	—	0%	0%
Case 7	0 volumes	—	—	—	0%	0%
Case 8	3 volumes	22 volumes	3 volumes	1 volume	95.5%	67%
Case 9	0 volumes	—	—	—	0%	0%
Case 10	4 volumes	22 volumes	4 volumes	1 volume	95.5%	75%
Case 11	3 volumes	22 volumes	3 volumes	1 volume	95.5%	67%
Case 12	1 volume	22 volumes	1 volume	1 volume	95.5%	0%
Case 13	2 volumes	22 volumes	2 volumes	1 volume	95.5%	50%
Case 14	1 volume	22 volumes	1 volume	1 volume	95.5%	0%
**Mean (all 14)**					**74.4%**	**35.8%**
**Mean (corrupted)**					**94.1%**	**50.1%**

TABLE S1 Acquisition cost comparison across the 14 prospective cases for three reacquisition strategies: conventional whole-acquisition repetition, conventional whole-volume repetition, and the proposed slice-level reacquisition.
